# Annoying Hiccups following Intra-Articular Corticosteroid Injection of Betamethasone Acetate/Betamethasone Sodium Phosphate at the Knee Joint

**DOI:** 10.1155/2013/829620

**Published:** 2013-03-14

**Authors:** George Habib, Suheil Artul, Geries Hakim

**Affiliations:** ^1^Rheumatology Clinic, Nazareth Hospital, 16000 Nazareth, Israel; ^2^Department of Medicine, Carmel Medical Center, 34362 Haifa, Israel; ^3^Radiology Department, Nazareth Hospital, 16000 Nazareth, Israel; ^4^Department of Orthopedics, Nazareth Hospital, 16000 Nazareth, Israel

## Abstract

Hiccups is a type of reflex that could happen secondary to different causes including drugs, especially systemic corticosteroids. Usually, high rather than regular doses of systemic steroids are incriminated, and this could explain the fact that very few cases of hiccups following regional corticosteroid treatment were reported. Here, we report the first case of hiccups in the English literature following intra-articular corticosteroid injection (IACI) at the knee joint and review all the previous reported cases of hiccups following regional corticosteroid treatment. Usually, this phenomenon of hiccups responds to regular antihiccups treatment; however, it is recommended not to repeat an IACI in a patient who had this adverse effect before due to an expected severe recurrent attack of hiccups afterwards.

## 1. Introduction

Hiccups is a reflex of a sudden contraction of the diaphragm and inspiration abruptly terminated by glottis closure. The mechanism behind this phenomenon is the stimulation of the hiccups reflex arc. This arc starts (afferent limb) with phrenic and vagus nerves and ends with the phrenic nerve again, glottis, and accessory muscles (efferent limb). Higher levels of the central nervous system like brain stem and midbrain affect this arc [1].

Hiccups could be induced by different etiologies including drugs, especially steroids [2]. It is believed that steroids trigger this reflex through steroid receptors on the reflex arc [3].

Intra-articular corticosteroid injection (IACI) is a common procedure and has a wide spectrum of systemic effects. Yet, there are very few reports of hiccups following regional steroid injections [4–7] including 3 cases only following IACI [5–7]. 2 of these cases were at the shoulder joint and one at the ankle joint. The knee joint is the most injected joint. To the best of our knowledge, hiccups following IACI at the knee joint was not reported before at the English literature.

 Here, we report a case of hiccups following IACI at the knee joint and review all the cases of hiccups following regional steroid injection that were reported or abstracted in English.

## 2. Case Presentation

42-year-old-male who works as a carpenter and started complains of bilateral knee pain, mostly during climbing-up or walking down the stairs. Serology was negative and also X-rays of the knees. MRI studies showed mild degenerative changes involving the cartilage and menisci. Following intolerance to nonsteroidal antiinflammatory drugs (NSAIDs) due to dyspepsia, he received an IACI of betamethasone acetate (3 mg)/betamethasone sodium phosphate (3 mg) (Celestone Chronodose, Schering-Plough, NV Belgium). Nearly 9 hours later, he started to feel an annoying hiccups preventing him from sleeping. A week later, omeprazole and metoclopramide were started with resolution of the symptoms within 3 days. Three weeks later patient received an intra-articular hyaluronic acid injection at the same knee joint (Suplasyn, Bioniche, Ireland) with favorable clinical response in terms of knee pain. Hiccups were not reported by the patient on followup.

## 3. Discussion

Hiccups following regional injections of corticosteroids including IACI are very rare. It is possible that the hiccups in our case occurred by chance and were unrelated to the IACI. However, the temporal relationship and existing data in the literature support this association. All the cases of hiccups following regional corticosteroid injection belonged to males (Table 1). In fact, most cases following intravenous treatment were also to males, suggesting an important role for sex hormones in the pathogenesis. Among the regional cases, 3 were 2 brothers and a nephew suggesting also a genetic role. All types of corticosteroids were used suggesting a class effect. The earliest time of the appearance of hiccups following the regional injection was 4 hours (glenohumeral joint). In 3 cases including our case, the hiccups were very disturbing. Hiccups lasted from 1 to 10 days; in some days it was self-limited and in others required regular antihiccup treatment with good response. In two cases, regional injection was repeated followed by recurrence of hiccups, even 6 years following the first episode, supporting the possibility of inherent tendency to develop hiccups. Recurrent hiccups were severe; in one case lasted for 2 weeks and in the other necessitated admission to the hospital. Intra-articular hyaluronic acid injection in two cases, were uneventful indicating that the hiccups were most probably related to the steroid compound in the injection.

## Figures and Tables

**Table 1:** Demographic and clinical parameters of the patients.

Parameter	Case number					
	1°	2°	3^±^	4′′	5^'^	6*
Age	52	48	38	42	66	42
Gender	Male	Male	Male	Male	Male	Male
Area of injection	Subacromial bursa	Plantar fascia	Ankle joint	Shoulder joint	Shoulder joint	Knee joint
Type of preparation	TA	MPA	BM	BM	Dexa	BM
Dose	NA	NA	NA	6 mg	NA	6 mg
Time of appearance of hiccups	Next day	NA	Next day	12 h	4 h	9 h
Duration of hiccups	72 h	24 h	24 h	1 day	2 days	10 days
Treatment and effect	Chlorpromazine Good	NA	None	Valsalva maneuver Good	NA	Metoclopramide Good
Recurrent IACI and type	Yes TA	No	Yes, TA	No	No	No
Recurrence of hiccups	Yes		Yes			
Time of appearance	NA		Next day			
Duration	NA		2 weeks			
Treatment and effect	Admission to hospital		Levomepromazine Good			

*Our case, °refrence number [4], ^±^reference number [5], ′′reference number [6], and ^'^reference number [7].

Abbreviations. IM: intramuscular, TA: triamcinolone preparation, MPA: methylprednisolone acetate, BM: betamethasone preparation, Dexa: dexamethasone preparation, NA: not available, and h: hour.
